# Stroke with neuropsychiatric sequelae after cannabis use in a man: a case report

**DOI:** 10.1186/1752-1947-5-264

**Published:** 2011-06-30

**Authors:** Benoit Trojak, Stéphanie Leclerq, Vincent Meille, Catia Khoumri, Jean-Christophe Chauvet-Gelinier, Maurice Giroud, Bernard Bonin, André Gisselmann

**Affiliations:** 1 Department of Psychiatry and Addictology, University Hospital of Dijon, 3 rue du Faubourg Raines, B.P. 1519, F-21033 Dijon Cedex, France; 2 Department of Neurology, University Hospital of Dijon, F-21033 Dijon Cedex, France

## Abstract

**Introduction:**

The outcome of cerebral ischemic stroke associated with cannabis use is usually favorable. Here we report the first case of cannabis-related stroke followed by neuropsychiatric sequelae.

**Case presentation:**

A 24-year-old Caucasian man was discovered in a deeply comatose non-reactive state after cannabis use. A magnetic resonance imaging scan of his brain showed bilateral multiple ischemic infarcts. The patient remained deeply comatose for four days, after which time he developed other behavioral impairments and recurrent seizures.

**Conclusion:**

Stroke related to cannabis use can be followed by severe neuropsychiatric sequelae. Concomitant alcohol intoxication is essential neither to the occurrence of this neurologic event nor to its severity.

## Introduction

Over the past few years, ischemic stroke associated with cannabis use has been reported in the literature. Typically, this accident concerns young, frequent cannabis smokers and usually occurs following cannabis consumption with simultaneous intake of alcohol, which is also thought to play a role in the cerebrovascular event [1]. In most case reports, the outcome of the neurovascular event was favorable and the patients rapidly recovered from stroke within hours or a few days.

Here we report the case of a young man who presented to our hospital with stroke that led to four days of deep coma, followed by neuropsychiatric sequelae. Moreover, this stroke occurred in the absence of alcohol intoxication.

### Case presentation

A 24-year-old Caucasian French man with no specific medical history was discovered in a deeply comatose, non-reactive state approximately 12 hours after he had fallen from a first-floor balcony under unknown circumstances. His blood pressure was 110/70 mmHg. During the physical examination, the mobile medical emergency team observed conjugate deviation of the eyes and concluded that the patient was having seizures. He was immediately treated with intravenous diazepam 10 mg, which had no impact on his level of consciousness, so he was intubated while on his way to our hospital.

The initial medical check-up conducted on admission to the medical intensive care unit did not reveal any biological anomalies related to his coma (electrolytes, glucose, ammonia level, liver and renal function tests, as well as arterial blood gas and carboxyhemoglobin levels). His electrocardiogram (ECG) and serum troponin I level were normal. His whole-body computed tomographic (CT) scan revealed thorax injuries due to the fall and excluded dissection of either the abdominal or thoracic aorta. His cerebral CT scan was unremarkable. Electroencephalography (EEG) (spot 20-minute recording) showed bilateral triphasic slow waves. Alcohol intoxication was excluded on the basis of a normal blood alcohol level. Urine toxicology (including tests for opioids, cocaine, amphetamines and psychotropic drugs) were negative except for the benzodiazepines administered by the emergency team before his hospital admission and for cannabis.

**Figure 1:**
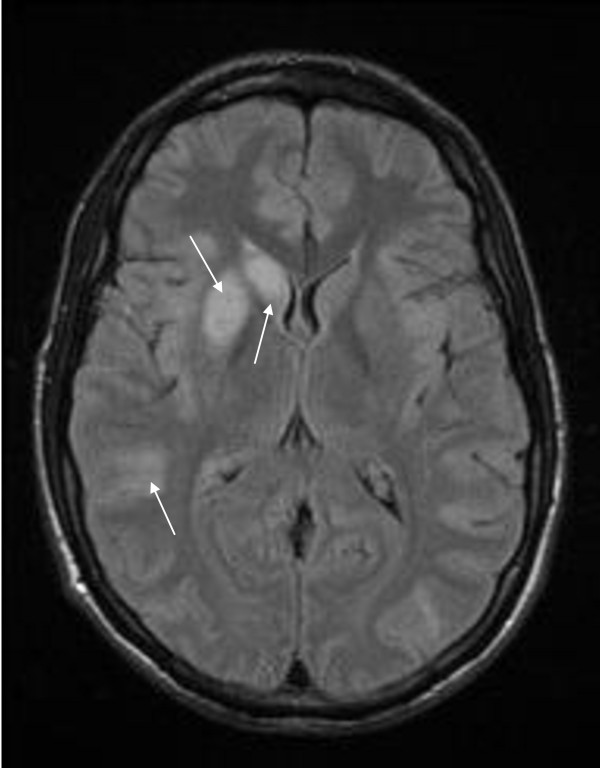
**T2-weighted magnetic resonance imaging scan showing bilateral superficial and deep to the right ischemic infarcts which concern different vascular territories in a young adult four days after he smoked cannabis**.

As the patient had not regained consciouness by day four, magnetic resonance imaging (MRI) of his brain was perfomed and revealed infarcts in the insular mantle and the lenticular and caudate nuclear structures (Figure 1), which were not consistent with traumatic contusions. A thorough evaluation did not reveal the cause of the stroke. EEG showed generalized slowing but did not give information on the patient's status epilepticus. Unfortunately, a transesophageal echocardiogram could not be performed secondary to the patient's behavioral condition. However, other studies were performed to help exclude cardioembolism, including ECG and transthoracic echocardiography. The examinations also excluded large-artery atherosclerosis (Doppler examination, magnetic resonance angiography and angiography scan) and hematological disorders (deficiencies in C and S proteins, resistance to activated C protein, dysfibrinogenemia, hyperhomocysteinemia, elevated factor VIII and D-dimer). Other causes of stroke in young adults, such as infectious or immunological disorders, were also excluded on the basis of virology tests, lumbar puncture, circulating anti-coagulant antibodies, cryoglobulins and monoclonal gammopathy.

**Figure 2:**
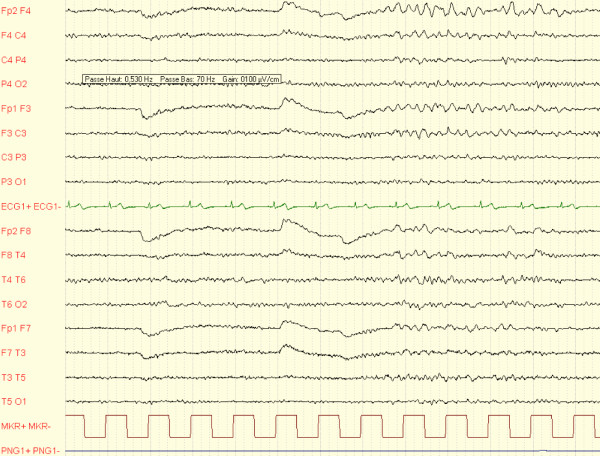
**Electroencephalographic (EEG) recording obtained within 24 hours after the patient's sixth generalized tonic-clonic seizure**. The EEG tracing shows brief pseudorhythmic θ activity that predominated bifrontally.

The patient had been a regular cannabis smoker (up to five cigarettes a day) for four years. According to the patient and his close relations, on the night before admission to our hospital, he had smoked more than 10 cannabis cigarettes. We thus concluded that the patient had experienced multiple arterial cerebral infarcts after cannabis use.

From day five, and for the following four weeks, the patient's cognitive function slowly improved, but he still presented behavioral disorders, with a loss of social awareness, sexual disinhibition manifesting as masturbation and genital exposure, emotional instability and impulsiveness. He was therefore referred to the psychiatric department for one month and was treated with an anti-psychotic (levomepromazine). He discharged himself from our hospital as a result of lack of judgment, blunted affect and poor insight. During the one and a half years following this hospitalization, the patient was readmitted to the hospital on seven occasions because of the occurrence of generalized tonic-clonic seizures. The patient received valproic acid as the anti-epileptic drug, but his adherence to treatment was probably poor. The patient did not exhibit symptoms of another stroke, even though he admitted that he occasionally smoked cannabis.

## Discussion

A recent review of the literature revealed 15 cases of stroke related to cannabis use, involving different arterial territories [2]. Three observations concerned cerebellar infarction in adolescent boys, which were fatal in two cases, and a much less severe infarction for the third, who recovered relatively well after his cerebellar stroke, since he presented with only mild dysdiadochokinesia in his right hand several weeks later [3]. The 12 other reported observations concerned cerebral stroke in young men. Contrary to our case report, the outcomes of these strokes were favorable for the majority of the patients, all of whom rapidly recovered from the cerebrovascular event except for a 22-year-old man who presented residual left-sided weakness after severe left hemiparesis [3]. Unlike our patient, none of the reported cases of cannabis-related stroke involved severe neuropsychiatric sequelae. As a consequence of his stroke, our patient now has cognitive impairment with behavioral disorders and recurrent seizures, and there may be a link between these three sequelae. Indeed, in the literature, a similar case of frontal syndrome with sexual disorders related to anterior cerebral infarction has been reported [4]. The risk of seizure in patients with lacunar infarct seems to be more dependent on the degree of cognitive impairment than on the severity of the stroke [5]. It has been suggested that the seizures are due not to lacunar infarcts but more probably to neurodegenerative processes that are also responsible for mental deterioration [5].

Since the latest case report by Mateo *et al. *[1], which described the case of a patient who had recurrent strokes after cannabis use but recovered, the link between cannabis and stroke has become highly plausible [1,6]. However, none of the various mechanisms that have been proposed to explain the association between stroke and cannabis use is satisfactory. The hypothesis of cardioembolism related to the arrhythmic properties of cannabis is generally not confirmed by clinical findings. The hypothesis that cannabis may induce vasospasm easily explains the transient cerebrovascular event usually reported in these circumstances, but this mechanism has not been demonstrated [2]. Postural hypotension has also been suggested, but, as in our case, most of the described patients were normotensive. The latest hypothesis is either toxic or immune inflammatory vasculopathy induced by smoking cannabis [1]. Indeed, arteritis similar to Buerger's disease involving peripheral vessels has been described after cannabis use, but there have been no descriptions of cerebral vasculitis [2]. It has also been suggested that concomitant alcohol ingestion may contribute to the neurologic event [1]. However, our present case report provides evidence that concomitant alcohol intoxication is not essential to either the occurrence of severe stroke during cannabis use or its severity. There is thus a need to investigate other mechanisms that can explain how cannabis, the most widely used illicit drug in the world, may lead to stroke in some users.

## Conclusion

Stroke related to cannabis use can be followed by severe neuropsychiatric sequelae. Concomitant alcohol intoxication is not essential to the occurrence of this neurologic event or to its severity.

## Abbreviations

CT: computed tomography; ECG: electrocardiogram; MRI: magnetic resonance imaging.

## Consent

Written informed consent was obtained from the patient for publication of this case report and any accompanying images. A copy of the written consent is available for review by the Editor-in-Chief of this journal.

## Competing interests

The authors declare that they have no competing interests.

## Authors' contributions

BT, MG and AG were involved in patient care and writing the report. SL, VM, CK, JCCG and BB participated in discussions and assisted in revising the report. All authors read and approved the final version of the manuscript.
